# The Chain Mediating Roles of Professional Identity and Workplace Social Capital in the Relationship Between Inclusive Leadership and Burnout of Nurses: A Longitudinal Study

**DOI:** 10.1155/jonm/4713030

**Published:** 2025-04-25

**Authors:** Yifan Wu, Wenwen Chen, Ruihong Zhang, Yang Yang, Hui Wang, Yang Xu, Shuang Zang

**Affiliations:** ^1^Department of Community Nursing, School of Nursing, China Medical University, Shenyang, China; ^2^Department of Basic Nursing, School of Nursing, Jilin University, Changchun, China; ^3^School of Politics and Public Administration, Wuhan University, Wuhan, China; ^4^Research and Education Department, Baoding No. 4 Central Hospital, Baoding, China; ^5^School of Nursing and Rehabilitation, Cheeloo College of Medicine, Shandong University, Jinan, China; ^6^Department of Radiology, The First Hospital of China Medical University, Shenyang, China

**Keywords:** burnout, inclusive leadership, nurses, professional identity, workplace social capital

## Abstract

**Background:** Burnout not only affects the health and work efficiency of nurses but also poses a potential threat to patient safety. The relationship and longitudinal mechanisms between inclusive leadership and nurse burnout in nursing work environments are unclear.

**Objective:** To analyze the pathways and mechanisms by which inclusive leadership influences nurse burnout through a longitudinal study and to explore the mediating role of professional identity and workplace social capital in this association.

**Method:** A two-wave study was conducted among 360 Chinese nurses from Baoding No. 4 Central Hospital in March 2024 and October 2024, respectively. The self-report questionnaire consisted of inclusive leadership scale, professional identity scale, workplace social capital scale, and Maslach Burnout Inventory. Pearson's correlation analysis was employed to explore the relationships among study variables. The structural equation modeling was applied to test the proposed model.

**Results:** The scores for inclusive leadership, professional identity, workplace social capital, and burnout among nurses were 36.35 ± 7.04, 37.80 ± 6.96, 33.22 ± 5.31, and 30.03 ± 14.23, respectively. There is a significant correlation between the above study variables (*p* < 0.001). Inclusive leadership negatively influenced burnout directly and indirectly through professional identity and workplace social capital (all *p* < 0.001). The indirect effects accounted for 65.58% of the total effect, with significant mediation through both pathways.

**Conclusions:** Inclusive leadership contributed to the elimination of burnout in nurses at work. In addition, professional identity and workplace social capital had chain mediation roles between the effects of the inclusive leadership and burnout.

**Implications for Nursing Management:** Nursing mangers should actively adopt an inclusive leadership to improve nurses' professional identity and workplace social capital to ameliorate burnout among clinical nurses.

## 1. Introduction

With the continuous development of the medical industry, clinical nurses are facing more and more pressure and challenges. In this high-pressure environment, burnout has become an increasingly serious problem. A latest meta-analysis shows that about 30.7% of nurses worldwide suffer from burnout [1]. Burnout is a psychological syndrome caused by long-term work pressure, which can cause nurses to lose enthusiasm and interest in their work, doubt the meaning and value of their work, and even deny their own ability to work [2]. Burnout not only affects the health and work efficiency of nurses but also poses a potential threat to treatment quality and patient safety [3]. It is therefore imperative to develop sustainable strategies to prevent burnout problems in nurses.

Although the causes of burnout are multifaceted, organizational and management issues are the most common causes of nurse burnout [4]. In particular, nurse leadership may be the key to preventing burnout [5]. Among various styles of nurse leadership, inclusive leadership is a leadership style that is open, available, and accessible when interacting with subordinates [6]. Inclusive leaders are willing to listen to employees' opinions, encourage subordinates to voice bold suggestions, tolerate subordinates' mistakes and omissions, and provide them with effective support when necessary [7]. A survey of 2299 registered nurses in China found that inclusive leadership could enhance nurses' psychological ownership, thereby buffering burnout under the normalization of COVID-19 prevention [8]. Another cross-sectional study targeting ICU nurses also indicated that inclusive leadership affected nurses' turnover intention, with burnout potentially playing a significant role in this relationship [9]. Although there is a close relationship between inclusive leadership and burnout, previous research has not disentangled the unique contribution of inclusive leadership to nurse burnout. Furthermore, these studies have mainly focused on the impact of inclusive leadership on nurse burnout in specific work environments, such as post-pandemic and ICU settings, almost neglecting the impact of inclusive leadership on nurse burnout in routine nursing environments and the potential differences in effects.

In addition, most studies on inclusive leadership among nurses are based on the perspective of social exchange theory, exploring the exchange relationship between leaders and their subordinates in the short term [10, 11]. However, previous research has shown that inclusive leadership preferred to minimize tension and allow things to change slowly [12]. Therefore, the actual impact of inclusive leadership may be difficult to identify through social exchange theory in a short period of time. Conservation of resources theory emphasizes the long-term impact of resource loss on individual mental health [13]. The theory holds that individuals strive to obtain, maintain, and protect their personal resources and social resources to achieve their goals, and the loss of resources is the main cause of stress and negative emotional reactions in individuals [14]. Long-term resource loss may lead to symptoms of burnout in individuals, such as emotional exhaustion, depersonalization, and reduced personal accomplishment [15]. Inclusive leadership helps to continuously protect and increase these resources, thereby reducing burnout [16].

According to the conservation of resources theory, professional identity, as a typical personal resource, can be a possible mediator in the relationship between inclusive leadership and burnout. Professional identity refers to positive cognition and emotional investment in the profession, which includes recognition of the value of work and the commitment to responsibilities [17]. Regarding inclusive leadership and professional identity, a cross-sectional study from Vietnam showed that inclusive leadership could create a supportive working atmosphere and enhance the professional identity of accountants [18]. Inclusive leadership could also fully mobilize medical staffs at different levels to meaningfully participate in medical activities and successfully promote their professional identity [19]. On the other hand, regarding professional identity and burnout, researchers emphasized that professional identity is an important protective factor against burnout among nurses [20]. Previous studies have shown that nurses with higher professional identity are less likely to suffer from burnout [21]. A systematic review of 30 studies also confirmed that the professional identity development program might avoid experiencing burnout caused by emotional stress [22]. Therefore, based on the above studies, inclusive leadership may reduce nurse burnout through professional identity.

Likewise, workplace social capital, as a social resource in conservation of resources theory, may mediate the relationship between inclusive leadership and burnout. Workplace social capital refers to the resources and support that individuals gain through their social networks in the workplace [23]. For nurses, social capital includes trust, cooperation, and information sharing among colleagues [24]. Numerous studies have shown that inclusive leadership is positively correlated with workplace social capital [25, 26]. A multilevel framework of inclusive leadership in organizations pointed out that inclusive leadership could promote collective-level interactions within a diverse work context and enhance workplace social capital [27]. A study of 325 corporate employees found that inclusive leadership may stimulate knowledge sharing behaviors to promote the workplace social capital [28]. Furthermore, workplace social capital is a key predictor of burnout. A longitudinal study suggested that workplace social capital might serve to prevent employee burnout [29]. Nursing practitioners are more dependent on workplace social capital to increase work engagement and thus regulate burnout [30, 31]. Therefore, given the above research, inclusive leadership may influence burnout through workplace social capital.

Professional identity, as an individual's psychological attribute to work, has an important impact on the formation and maintenance of workplace social capital. A study of college students from two institutions showed that the strength of professional identity affected students' acquisition of other key resources after entering the labor market, especially workplace social capital [32]. In addition, research showed that nurses' professional identity could facilitate their building of stronger workplace social capital, as nurses with a strong sense of identity were more likely to actively participate in team activities and build trusting relationships with colleagues. According to social identity theory, individual identification with the group is the basis of group behavior [33]. In other words, nurses' professional identity helps to enhance their behavior and willingness to maintain workplace social capital [34]. Therefore, professional identity may affect workplace social capital.

**Figure 1:**
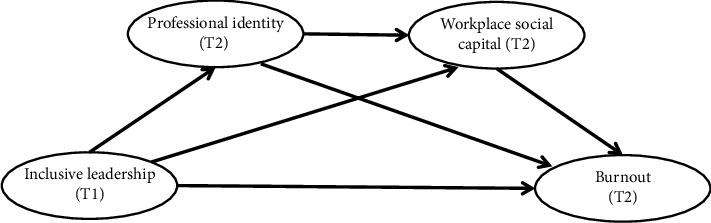
The hypothesized study model. Note: T1 = time 1, T2 = time 2.

In summary, professional identity and workplace social capital may be involved in the mechanism pathway of inclusive leadership's impact on burnout. However, how these variables affect each other has not been explored and confirmed. Moreover, it is difficult to identify the actual impact of inclusive leadership in a short period of time because of the underlying slow change it brings. At the same time, professional identity, workplace social capital, and nurses' burnout are also dynamic processes that can be continuously developed and reconstructed [35, 36]. Most of the previous studies in related fields are cross-sectional designs, and there is still a lack of longitudinal studies with multiple measurements and stronger causal explanations. Therefore, disentangling the roles of inclusive leadership, professional identity, and workplace social capital in burnout is essential for informing interventions aimed at preventing medical quality issues caused by burnout among nurses. This study aims to use longitudinal data to explore the dynamic relationship between inclusive leadership and burnout, and further examine the mediating role of professional identity and workplace social capital in this process. The research hypotheses of this study are as follows (Figure 1):

  Hypothesis 1. Inclusive leadership contributes to the elimination of burnout in nurses at work.
  Hypothesis 2. Professional identity mediates the relationship between inclusive leadership and burnout in nurses.
  Hypothesis 3. Workplace social capital mediates the relationship between inclusive leadership and burnout in nurses.
  Hypothesis 4. Professional identity and workplace social capital have a chain mediating effect between inclusive leadership and burnout in nurses.

## 2. Methods

### 2.1. Participants and Procedure

Participants in this longitudinal study were clinical nurses at Baoding No. 4 Central Hospital in Hebei Province, China. Eligibility for participation was based on the following criteria: (i) being a registered nurse, (ii) being a regular employee of the hospital, and (iii) having practiced nursing for at least 6 months. Exclusion criteria included: (i) nurses who were on sick leave, maternity leave, or away on training and (ii) those participating in other similar research projects.

After obtaining approval from the management of the hospital, the questionnaires were delivered by the researchers to the hospital. Subsequently, the hospital nursing administration distributed the questionnaires to the nurses. Along with the questionnaires, detailed instructions on how to fill them in were provided to ensure clarity and accuracy in the responses. Data were collected in two waves with 6-month intervals: March 2024 (T1) and October 2024 (T2). Demographic information and inclusive leadership were measured by self-reported questionnaires at T1. Professional identity, workplace social capital, and nurse burnout were measured at T2.

The sample size was calculated based on the formula for single-group repeated measures: *M*=([1+(*k* − 1)*ρ*]*σ*^2^(*Z*_*α*/2_+*Z*_*β*_)^2^/*kδ*^2^), where *α* = 0.05, *Z*_*α*/2_ = 1.96, *β = *0.20, *Z*_*β*_ = 0.84, and the number of repeated measurements *k* = 2 in this study. According to existing studies [16, 37], we set *ρ* = −0.41, *σ* = 0.62, and *δ* = 0.159. We also considered a 20% dropout rate, a minimum of 331 participants is required. Finally, a total of 474 participants provided valid information in the baseline survey at T1, and 360 (75.95%) remained at T2. Data from the second wave were matched to the previous wave by the specific encoding.

This project was approved by the Ethics Committee of the Baoding No. 4 Central Hospital (approval number: 2024007). Informed consent was obtained from all participants involved in the study. The consent process included a detailed explanation of the study's objectives, procedures, potential benefits, and risks. Participants had the right to withdraw from the study at any time without any consequences. Confidentiality was strictly maintained throughout the study, with all data being anonymized and coded to ensure that individual responses could not be linked back to the participants. Personal identifiers were removed from the data set, and only authorized researchers had access to the coded data. The data collected were used exclusively for research data analysis.

### 2.2. Measures

#### 2.2.1. Demographic Information

The demographic information questionnaire encompassed several demographic and professional variables. Age was included as a continuous variable. Gender (male, female), type of employment (contract, permanent, or other), education level (high school or technical secondary, bachelor, master, or above), professional title (junior, intermediate, vice-senior, or senior), being a leader or not (yes, no), length of service (< 5, 6–10, 11–15, 16–20, and > 20), and frequency of night shifts per week (0, 1, 2, and ≥ 3) were included as categorical variables.

#### 2.2.2. Inclusive Leadership

The inclusive leadership scale was employed to measure inclusive leadership [38]. The scale includes three dimensions of openness (three items), accessibility (two items), and availability (four items), with a total of nine items. Each item is rated on a five-point Likert scale, with scores ranging from 1 = “strongly disagree” to 5 = “strongly agree.” A higher total score indicates a higher level of inclusive leadership. In this study, the Cronbach's α for the inclusive leadership scale was 0.969.

#### 2.2.3. Professional Identity

The professional identity scale was used to measure professional identity [39]. The scale consists of 10 items. Each item is rated on a five-point Likert scale, with scores ranging from 1 = “strongly disagree” to 5 = “strongly agree.” A higher total score indicates a higher level of professional identity. In this study, the Cronbach's alpha for the professional identity scale was 0.944.

#### 2.2.4. Workplace Social Capital

The workplace social capital scale was employed to measure workplace social capital [40]. The scale consists of cognitive dimension (three items) and structural dimension (five items), with a total of eight items. Each item is rated on a five-point Likert scale, with scores ranging from 1 = “strongly disagree” to 5 = “strongly agree.” A higher total score indicates a higher level of workplace social capital. In this study, the Cronbach's α for the workplace social capital scale was 0.970.

#### 2.2.5. Burnout

The Maslach burnout inventory was utilized to measure burnout [41]. The scale consists of three dimensions of emotional exhaustion (five items), depersonalization (four items), and personal accomplishment (six items), with a total of fifteen items. Each item is scored on a seven-point Likert scale ranging from 0 = “never” to 6 = “every day.” The personal accomplishment dimension is reverse-scored. The higher the total score after reverse scoring, the higher the level of burnout. In this study, the Cronbach's alpha for the Maslach burnout inventory was 0.920.

### 2.3. Data Analysis

Statistical analyses were performed using the IBM SPSS 25.0 (SPSS Inc. Chicago, IL, USA) and AMOS 24.0 (IBM Corp., Armonk, NY, USA) software. Initially, the normality of the data distribution was evaluated using the Kolmogorov–Smirnov test and Q-Q plots. All continuous variables exhibited an approximately normal distribution. Categorical variables were described by numbers with percentages and continuous variables being described by means of standard deviation (SD). Subsequently, bivariate Pearson's correlations were used to examine the relationships between the study variables. Furthermore, the model fit indices and mediation effects were analyzed through structural equation modeling. All models were evaluated using standardized coefficients derived from maximum likelihood. The bias-corrected nonparametric percentile Bootstrap method (5000 resamples) was applied to estimate the 95% confidence interval (CI) for the indirect effects of mediators. A well-fitting model was determined by the following criteria: *χ*^2^/df was < 3.00, and the value of RMSEA was < 0.08, while values of CFI, GFI, AGFI, NFI, IFI, and TLI were all > 0.90 [42]. A *p* value of less than 0.05 was considered statistically significant.

## 3. Results

### 3.1. Demographic Characteristics

The mean age of the participants was 32.61 ± 6.66 years (age range 22–60 years). Among them, 351 (97.50%) were females, 187 (51.94%) were contract nurses, and the education level was predominantly bachelor's degree (69.17%). The total number of junior nurses and leaders were 178 (49.44%) and 59 (16.11%), respectively. In terms of length of service, 196 (54.44%) participants had worked for more than 10 years. Most nurses (83.05%) had less than two night shifts per week. More details for demographic characteristics were shown in Table 1.

### 3.2. Descriptive Analysis and Correlation Analysis

The scores for inclusive leadership (T1), professional identity (T2), and workplace social capital (T2) among nurses were 36.35 ± 7.04, 37.80 ± 6.96, and 33.22 ± 5.31, respectively, which were all at a moderately high level. The burnout (T2) score of 30.03 ± 14.23 indicated that burnout among nurses was at a low level (Table 2). The results for all correlations were presented in Table 3. The results indicated that inclusive leadership (T1) was significantly positively correlated with professional identity (T2) (*r* = 0.491, *p* < 0.001) and workplace social capital (T2) (*r* = 0.490, *p* < 0.001), and significantly negatively correlated with burnout (T2) (*r* = −0.512, *p* < 0.001). Burnout was strongly negatively correlated with both professional identity (T2) (*r* = −0.659, *p* < 0.001) and workplace social capital (T2) (*r* = −0.623, *p* < 0.001). Professional identity was also highly correlated with workplace social capital (T2) (*r* = 0.669, *p* < 0.001).

### 3.3. Analysis of Mediation Effects

The structural equation model showed acceptable goodness-of-fit indices: *χ*^2^ = 59.207, df = 22, *χ*^2^*/*df = 2.691, CFI = 0.985, GFI = 0.964, AGFI = 0.927, NFI = 0.976, IFI = 0.985, TLI = 0.975, and RMSEA = 0.069. These results indicated that the mediation model for inclusive leadership (T1) and burnout (T2) among nurses is reasonable.

**Figure 2:**
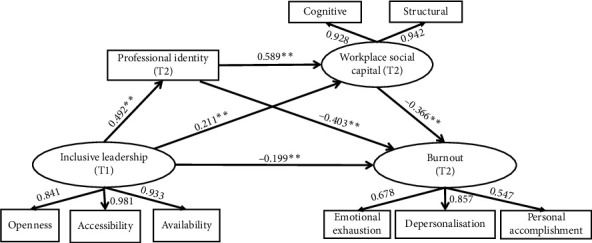
The chain mediating effect of workplace social capital (T2) and workplace social capital (T2) on the chain between inclusive leadership (T1) and burnout. Note: T1 = time 1, T2 = time 2, ^∗∗^*p* < 0.001 (two-tailed).

The standardized path coefficient model depicted in Figure 2 revealed that inclusive leadership (T1) contributed to the elimination of burnout (T2) in nurses at work, with a path coefficient of *β* = −0.199 (*p* < 0.001), thereby supporting Hypothesis 1. Furthermore, inclusive leadership (T1) significantly impacted professional identity (T2) (*β* = 0.492, *p* < 0.001), which in turn significantly impacted burnout (T2) (*β* = −0.403, *p* < 0.001), confirming that professional identity (T2) mediated the relationship between inclusive leadership (T1) and burnout (T2), thus supporting Hypothesis 2. In addition, inclusive leadership (T1) significantly influenced workplace social capital (T2) (*β* = 0.211, *p* < 0.001), and workplace social capital (T2) significantly influenced burnout (T2) (*β* = −0.366, *p* < 0.001), indicating that workplace social capital (T2) mediated the relationship between inclusive leadership (T1) and burnout (T2), supporting Hypothesis 3. Professional identity also exerted a simultaneous significant and positive effect on workplace social capital (T2) (*β* = 0.589, *p* < 0.001). Therefore, there was a chain mediating effect of professional identity (T2) and workplace social capital (T2) between inclusive leadership (T1) and burnout (T2), which supported Hypothesis 4. The detailed results of the mediated effects analysis were presented in Table 4.

Table 5 showed the total, direct, and indirect effects of inclusive leadership (T1), professional identity (T2), and workplace social capital (T2) on burnout (T2). The direct effect of inclusive leadership (T1) on nurses' burnout (T2) was significant (direct effect = −0.199, 95% CI [−0.295, −0.098]), accounting for 34.25% of the total effect. The mediating effect of professional identity (T2) and workplace social capital (T2) was significant, with a total indirect effect value of −0.381 (95% CI [−0.467, −0.306]), constituting 65.58% of the total effect. The indirect effects included three significant mediation pathways: first, inclusive leadership (T1) indirectly affected burnout (T2) through professional identity (T2) (indirect effect = −0.198, 95% CI [−0.284, −0.126]). Second, inclusive leadership (T1) indirectly affected burnout (T2) through workplace social capital (T2) (indirect effect = −0.077, 95% CI [−0.141, −0.037]). Third, inclusive leadership (T1) indirectly affected burnout (T2) through both professional identity (T2) and workplace social capital (T2) (indirect effect = −0.106, 95% CI [−0.164, −0.065]).

## 4. Discussion

To the best of our knowledge, this is the first study to examine the role and pathways of inclusive leadership on burnout among Chinese clinical nurses. The longitudinal study design provides a profound understanding of the dynamic relationships between study variables. This study found that inclusive leadership contributed to the elimination of burnout in nurses at work. In addition, professional identity and workplace social capital had chain mediation roles between the effects of inclusive leadership and burnout.

In this study, inclusive leadership was at a moderately high level, with a mean score of 36.35 ± 7.04 (on a scale from 9 to 45), demonstrating nursing leaders' tendency to be inclusive towards nurses. This finding was close to the results of a Chinese study measuring inclusive leadership among nursing leaders in tertiary hospitals, which reported a mean score of 35.49 ± 3.68 (on a similar scale) [43]. This phenomenon may be explained by the fact that nursing leaders in tertiary hospitals have a relatively high level of education and demonstrate appropriate inclusiveness at the managerial level. Such similarity also reflected the prevalence of nurses' perception of inclusive leadership. In addition, this study found that burnout among nurses was at a low level. A study of burnout among nursing staff at a tertiary general hospital in Shanghai, China, yielded results similar to the personal accomplishment scores in this study but scored lower on the emotional exhaustion and depersonalization dimensions [44]. It might suggest that the nurses' low burnout relied heavily on personal accomplishment to sustain. This also hinted at the potential protective role of inclusive leadership in the emotional exhaustion and depersonalization dimensions.

This study found that inclusive leadership was more likely to prevent nurses from experiencing burnout. This finding parallels the research of Leclerc et al., who emphasized the protective effect of relational leadership on nurse burnout, reporting a reduction in turnover from 60% to 15% among nurses under relational leadership [45]. The difference is that our study focused on the specific leadership style of inclusive leadership – the core of relational leadership. One of the potential mechanisms by which inclusive leadership impacts burnout can be explained by psychological safety [46]. Inclusive leadership allows their subordinates to fail and will actively guide their subordinates to rationalize their personal strengths. In this process, the psychological safety brought by inclusive leadership may effectively reduce employees' uncertainty and stress, thus reducing burnout [16]. In addition, in the Chinese context, inclusive leadership has been endowed with the moral quality of acceptance, which has been developed as the core connotation of Confucianism's “benevolence” ideology [47]. Therefore, in China, inclusive leaders are more likely to be recognized in long-term interactions with subordinates. Such recognition helps to enhance nurses' job satisfaction, thereby reducing the risk of burnout [48]. Consequently, nursing leaders may consider the appropriate use of inclusive leadership strategies to help nurses cope with burnout. Previous studies have shown that effective strategies for inclusive leadership include providing psychological support, encouraging open communication, distributing resources fairly, and empowering employees with autonomy [49]. These strategies help promote team member well-being and work-life balance, thereby reducing the incidence of burnout [49].

This study revealed that professional identity mediates the impact of inclusive leadership on nurse burnout, which is consistent with existing research. A cross-sectional study showed that high levels of inclusive leadership tended to correspond with higher levels of professional identity [50]. Another study found that nurses with strong professional identity were more resistant to burnout, which is similar to the results of this study [51]. Our study reveals how inclusive leadership shapes professional identity over time, which in turn influences burnout. On the one hand, inclusive leadership can help nurses shape their professional identities by providing support and respect. Research has shown that nurses are more likely to feel valued in their profession when leaders demonstrate respect for individual differences [52]. Inclusive leadership can also promote nurses' professional growth and self-actualization in the nursing field, which can further enhance and solidify their professional identity [53]. On the other hand, according to the social identity theory, individuals tend to define themselves through their professional roles [54]. Professional identity reinforces nurses' professional roles, enabling them to resist stress and reduce burnout [55]. In addition, professional identity, as a part of self-concept, helps nurses to maintain a positive attitude in the face of challenges and consequently reduces burnout [56]. According to a meta-analysis of interventions to reduce burnout in nurses, the most effective interventions are those that provide a training approach, such as professional identity development programs [57]. This intervention should be actively supported by leadership to ensure its integration into the practice of individual nurses. Nursing managers can also hold regular one-on-one meetings with nurses to discuss their professional development and challenges, demonstrating a genuine concern for their well-being and professional growth [58].

The present study showed that inclusive leadership influences burnout through workplace social capital. This finding is consistent with existing research that emphasizes the role of social capital in reducing burnout [59]. In contrast, the present study provides deeper insights as we observed that inclusive leadership may have influenced workplace social capital 6 months later before influencing nurse burnout. The positive impact of inclusive leadership on workplace social capital has been widely demonstrated. Studies have shown that inclusive leadership encourages fair treatment and promotes trust and cooperation among coworkers, which are important components of workplace social capital [60]. At the same time, inclusive leadership ensures communication and knowledge sharing, which facilitates nurses to increase workplace social capital [61]. As for the buffering effect of workplace social capital on burnout, it can be explained by perceived organizational support theory. The theory suggests that the level of organizational support felt by employees affects their work attitudes and behaviors [62]. Studies have shown that nurses with higher workplace social capital are more likely to receive support from their coworkers, which helps them manage stress and diminish burnout [63]. Workplace social capital promotes teamwork, which also helps nurses cope with emotional exhaustion and reduce burnout. Therefore, creating a supportive work environment where nurses feel connected to their colleagues may significantly reduce their burnout. Previous studies have shown that structured team-building activities can significantly promote informal interactions, thereby increasing the density of social capital [64]. This suggests that healthcare institutions should regularly organize workshops and interest groups to create informal communication scenarios for nurses [65]. Research has also confirmed that cross-departmental collaboration platforms can enhance weak-tie networks within organizations [66]. It is recommended to establish departmental rotation programs or interdisciplinary project teams to expand the social support networks of nurses, which in turn can lead to a reduction in burnout among nurses [67].

The unique aspect of this study is the revelation of the chain-mediated effects of inclusive leadership on burnout through professional identity and workplace social capital. This provides a more complex chain of causality than previous studies. For example, the previous study emphasized the impact of professional identity on burnout [68], but our study further reveals the chain-mediating role of professional identity and workplace social capital in this process, which provides new perspectives for comprehending the causes of burnout. The effect of professional identity on workplace social capital is reflected in the fact that a strong professional identity can motivate employees to actively participate in social interactions in the workplace, thereby increasing workplace social capital [69]. The stronger the nurses' professional identity, the more likely they are to demonstrate professional commitment and willingness to share knowledge and resources with coworkers, thus enhancing workplace social capital [70]. Some researchers have pointed out that burnout is not just a mental health issue at the individual level but is closely related to workplace and organizational factors [71]. Therefore, it is not enough to rely only on the efforts of individuals to reduce burnout. There is a strong need to strengthen organizational-level support and interventions, such as inclusive leadership and workplace social capital, to more effectively prevent and reduce the occurrence of burnout.

In summary, inclusive leadership has both direct and indirect effects on nurse burnout. Notably, the indirect effect had a greater path weight than the direct effect, which emphasizes the importance of investigating the mechanisms by which inclusive leadership affects nurse burnout. The results of this study provide multiple pathways to reduce nurse burnout and provide quality nursing care. These include applying inclusive leadership, improving nurse professional identity, and strengthening workplace social capital. Future research should extend these findings to provide a more comprehensive understanding of how inclusive leadership affects nurse burnout. Compared to cross-sectional design in previous studies, our longitudinal study design can promote causal inference and more comprehensively investigate the direction of the associations between inclusive leadership and burnout. It is also necessary to periodically assess the impact of inclusive leadership, professional identity, and workplace social capital on burnout in order to make timely adjustments to management strategies.

## 5. Limitations

This study has several limitations. First, the self-reported measures may have introduced self-report bias and shared method variance. Second, this study was conducted in only one hospital in China, which may have limited the representativeness of the sample. Meanwhile, it is unclear whether the findings can be generalized to other cultural contexts. Third, because of practical constraints, the interval between the two waves of data in this study was short, only 6 months. While this interval allowed us to capture initial changes and trends, a longer time interval, such as 1 year, would be ideal and help to examine more accurate causal sequences in future investigations. Fourth, the possible influence of some other variables on nurse burnout cannot be ruled out, and more extensive research is needed to enrich the existing findings.

## 6. Conclusion

The findings of this study underscore the critical role of inclusive leadership in mitigating nurse burnout through its positive impact on professional identity and workplace social capital. Nursing leaders should actively adopt inclusive leadership practices as a strategic approach to address burnout. This includes providing regular feedback, recognizing individual contributions, and facilitating opportunities for professional development and teamwork. Future research should continue to explore the long-term effects of inclusive leadership on nurse well-being and organizational outcomes, as well as identify additional interventions that can further enhance the benefits of inclusive leadership in healthcare settings.

## Figures and Tables

**Table 1:** Demographic characteristics of participants at baseline (*N* = 360).

Variables	*N*	Percentage (%)
Age	32.61 ± 6.66 (mean ± SD)	

*Gender*		
Male	9	2.50
Female	351	97.50

*Type of employment*		
Contract	187	51.94
Permanent	148	41.11
Other	25	9.64

*Education level*		
High school or technical secondary	110	30.56
Bachelor	249	69.17
Master and above	1	0.28

*Professional title*		
Junior	178	49.44
Intermediate	140	38.89
Vice-senior	35	9.72
Senior	7	1.94

*Being a leader or not*		
Yes	59	16.11
No	302	83.89

*Length of service (years)*		
< 5	101	28.06
6–10	62	17.22
11–15	129	35.83
16–20	34	9.44
> 20	33	9.17

*Frequency of night shifts (per week)*		
0	98	27.22
1	103	28.61
2	98	27.22
≥ 3	61	16.94

*Note:* Percentages may not add up to 100% owing to rounding.

Abbreviation: SD = standard deviation.

**Table 2:** Descriptive analysis of study variables (*N* = 360).

Study variables	Min	Max	Mean ± SD
Inclusive leadership (T1)	11	45	36.35 ± 7.04
Openness (T1)	3	15	12.03 ± 2.45
Accessibility (T1)	2	10	16.18 ± 3.31
Availability (T1)	4	20	8.11 ± 1.66
Professional identity (T2)	13	50	37.80 ± 6.96
Workplace social capital (T2)	10	40	33.22 ± 5.31
Cognitive dimension (T2)	4	15	12.56 ± 2.04
Structural dimension (T2)	6	25	20.66 ± 3.43
Burnout (T2)	0	81	30.03 ± 14.23
Emotional exhaustion (T2)	0	30	11.29 ± 5.56
Depersonalization (T2)	0	24	5.82 ± 4.18
Personal accomplishment (T2)	0	29	12.93 ± 8.01

*Note:* Min = minimum, Max = maximum, T1 = time 1, T2 = time 2.

Abbreviation: SD = standard deviation.

**Table 3:** Correlation analysis of study variables (*N* = 360).

Number	Study variables	1	2	3	4	5	6	7	8	9	10	11	12
1	Inclusive leadership (T1)	1											
2	Openness (T1)	0.921^∗∗^	1										
3	Accessibility (T1)	0.941^∗∗^	0.789^∗∗^	1									
4	Availability (T1)	0.972^∗∗^	0.821^∗∗^	0.915^∗∗^	1								
5	Professional identity (T2)	0.491^∗∗^	0.471^∗∗^	0.409^∗∗^	0.490^∗∗^	1							
6	Workplace social capital (T2)	0.490^∗∗^	0.476^∗∗^	0.431^∗∗^	0.473^∗∗^	0.669^∗∗^	1						
7	Cognitive dimension (T2)	0.466^∗∗^	0.455^∗∗^	0.407^∗∗^	0.449^∗∗^	0.649^∗∗^	0.949^∗∗^	1					
8	Structural dimension (T2)	0.481^∗∗^	0.466^∗∗^	0.424^∗∗^	0.464^∗∗^	0.648^∗∗^	0.982^∗∗^	0.874^∗∗^	1				
9	Burnout (T2)	−0.512^∗∗^	−0.492^∗∗^	−0.440^∗∗^	−0.504^∗∗^	−0.659^∗∗^	−0.623^∗∗^	−0.602^∗∗^	−0.606^∗∗^	1			
10	Emotional exhaustion (T2)	−0.396^∗∗^	−0.363^∗∗^	−0.329^∗∗^	−0.408^∗∗^	−0.580^∗∗^	−0.485^∗∗^	−0.449^∗∗^	−0.483^∗∗^	0.760^∗∗^	1		
11	Depersonalization (T2)	−0.493^∗∗^	−0.479^∗∗^	−0.415^∗∗^	−0.486^∗∗^	−0.627^∗∗^	−0.616^∗∗^	−0.587^∗∗^	−0.604^∗∗^	0.828^∗∗^	0.688^∗∗^	1	
12	Personal accomplishment (T2)	−0.378^∗∗^	−0.372^∗∗^	−0.338^∗∗^	−0.359^∗∗^	−0.440^∗∗^	−0.448^∗∗^	−0.450^∗∗^	−0.426^∗∗^	0.817^∗∗^	0.296^∗∗^	0.471^∗∗^	1

*Note:* T1 = time 1, T2 = time 2.

^∗∗^ *p* < 0.001 (two-tailed).

**Table 4:** Regression analysis of the relationship among study variables in the mediation effect model.

Outcome variable	Predictor variable	*R* ^2^	*β*	SE	*t*	LLCI	ULCI
Professional identity (T2)	Inclusive leadership (T1)	0.242	0.492	0.043	10.258^∗∗^	0.397	0.576

Workplace social capital (T2)	Inclusive leadership (T1)	0.514	0.211	0.038	4.534^∗∗^	0.109	0.315
	Professional identity (T2)		0.589	0.043	12.38^∗∗^	0.482	0.682

Burnout (T2)	Inclusive leadership (T1)	0.692	−0.199	0.049	−3.844^∗∗^	−0.295	−0.098
	Professional identity (T2)		−0.403	0.072	−5.884^∗∗^	−0.542	−0.258
	Workplace social capital (T2)		−0.366	0.081	−5.228^∗∗^	−0.516	−0.221

*Note:* T1 = time 1, T2 = time 2.

Abbreviations: LLCI = low limit confidence interval, SE = standard error, ULCI = upper limit confidence interval.

^∗∗^ *p* < 0.001 (two-tailed).

**Table 5:** Total, direct, and indirect effects of inclusive leadership on burnout.

Influence path	Effect value	Boot SE	LLCI	ULCI	Relative mediation effect (%)
Total effect	−0.581	0.042	−0.664	−0.498	100
Direct effect	−0.199	0.050	−0.295	−0.098	34.25
Total indirect effect	−0.381	0.041	−0.467	−0.306	65.58
Inclusive leadership (T1) ⟶ professional identity (T2) ⟶ burnout (T2)	−0.198	0.040	−0.284	−0.126	51.97
Inclusive leadership (T1) ⟶ workplace social capital (T2) ⟶ burnout (T2)	−0.077	0.026	−0.141	−0.037	20.21
Inclusive leadership (T1) ⟶ professional identity (T2) ⟶ workplace social capital (T2) ⟶ burnout (T2)	−0.106	0.025	−0.164	−0.065	27.82

*Note:* T1 = time 1, T2 = time 2.

Abbreviations: LLCI = low limit confidence interval, SE = standard error, ULCI = upper limit confidence interval.

## Data Availability

The data that support the findings of this study are available from the corresponding author upon reasonable request.

## References

[1] Li L. Z., Yang P., Singer S. J., Pfeffer J., Mathur M. B., Shanafelt T. (2024). Nurse Burnout and Patient Safety, Satisfaction, and Quality of Care: A Systematic Review and Meta-Analysis. *JAMA Network Open*.

[2] Yuan C.-M., Xu C.-Y. (2020). Concept Analysis of Nurse Burnout. *Frontiers of Nursing*.

[3] Pérez-Francisco D. H., Duarte-Clíments G., del Rosario-Melián J. M., Gómez-Salgado J., Romero-Martín M., Sánchez-Gómez M. B. Influence of Workload on Primary Care Nurses’ Health and Burnout, Patients’ Safety, and Quality of Care: Integrative Review. *Healthcare*.

[4] Fallahi-Khoshknab M., Ghavidel F., Molavynejad S., Zarea K. (2019). The Role of Organizational Factors in Nurse Burnout: Experiences From Iranian Nurses Working in Psychiatric Wards. *Journal of Family Medicine and Primary Care*.

[5] Wei H., King A., Jiang Y., Sewell K. A., Lake D. M. (2020). The Impact of Nurse Leadership Styles on Nurse Burnout: A Systematic Literature Review. *Nurse Leader*.

[6] Roberson Q., Perry J. L. (2022). Inclusive Leadership in Thought and Action: A Thematic Analysis. *Group & Organization Management*.

[7] Veli Korkmaz A., Van Engen M. L., Knappert L., Schalk R. (2022). About and beyond Leading Uniqueness and Belongingness: A Systematic Review of Inclusive Leadership Research. *Human Resource Management Review*.

[8] Zeng D., Wang B., Chen W. (2022). Inclusive Leadership Can Improve Nurses’ Psychological Ownership and Reduce Their Turnover Intention under the Normalization of COVID-19 Prevention. *Frontiers in Psychology*.

[9] Du H., Huang H., Li D., Zhang X. (2024). The Effect of Inclusive Leadership on Turnover Intention of Intensive Care Unit Nurses: The Mediating Role of Organization-Based Self-Esteem and Interactional Justice. *BMC Nursing*.

[10] Qi L., Liu B., Wei X., Hu Y. (2019). Impact of Inclusive Leadership on Employee Innovative Behavior: Perceived Organizational Support as a Mediator. *PLoS One*.

[11] Brown B. K., Subramaniam C., Ali H. (2017). Inclusive Leadership, Safety Climate and Safety Behaviour: A Proposed Framework. *International Journal of Academic Research in Business and Social Sciences*.

[12] King S., Roberts-Turner R., Floyd T. T. (2024). Inclusive Leadership: A Framework to Advance Diversity, Equity, Inclusion, and Cultivate Belonging. *Nurse Leader*.

[13] Chen G., Wang J., Huang Q. (2024). Social Support, Psychological Capital, Multidimensional Job Burnout, and Turnover Intention of Primary Medical Staff: A Path Analysis Drawing on Conservation of Resources Theory. *Human Resources for Health*.

[14] Hobfoll S. E., Freedy J. (2017). Conservation of Resources: A General Stress Theory Applied to Burnout. *Professional Burnout*.

[15] Prapanjaroensin A., Patrician P. A., Vance D. E. (2017). Conservation of Resources Theory in Nurse Burnout and Patient Safety. *Journal of Advanced Nursing*.

[16] Li X., Peng P. (2022). How Does Inclusive Leadership Curb Workers’ Emotional Exhaustion? The Mediation of Caring Ethical Climate and Psychological Safety. *Frontiers in Psychology*.

[17] Fitzgerald A. (2020). Professional Identity: A Concept Analysis. *Nursing forum*.

[18] Huy P. Q., Phuc V. K. (2024). Unraveling Role of Accountants’ Professional Identity Toward Sustainable Development: Does Inclusive Leadership Make a Difference?. *International Journal of Disclosure and Governance*.

[19] Bradley E. H. (2020). Diversity, Inclusive Leadership, and Health Outcomes. *International Journal of Health Policy and Management*.

[20] Jiang H., Huang N., Jiang X., Yu J., Zhou Y., Pu H. (2021). Factors Related to Job Burnout Among Older Nurses in Guizhou Province, China. *PeerJ*.

[21] Goliroshan S., Nobahar M., Raeisdana N., Ebadinejad Z., Aziznejadroshan P. (2021). The Protective Role of Professional Self-Concept and Job Embeddedness on Nurses’ Burnout: Structural Equation Modeling. *BMC Nursing*.

[22] De Oliveira S. M., de Alcantara Sousa L. V., Vieira Gadelha M. d. S., do Nascimento V. B. (2019). Prevention Actions of Burnout Syndrome in Nurses: An Integrating Literature Review. *Clinical Practice and Epidemiology in Mental Health: CP & EMH*.

[23] Tsounis A., Xanthopoulou D., Demerouti E., Kafetsios K., Tsaousis I. (2023). Workplace Social Capital: Redefining and Measuring the Construct. *Social Indicators Research*.

[24] Jutengren G., Jaldestad E., Dellve L., Eriksson A. (2020). The Potential Importance of Social Capital and Job Crafting for Work Engagement and Job Satisfaction Among Health-Care Employees. *International Journal of Environmental Research and Public Health*.

[25] Li Z., Wang B., Shan Z., Zhu J. (2024). Integrated Human Resource Practices and Employee Creativity: The Roles of Social Capital, Human Capital and Inclusive Leadership. *Transformations in Business and Economics*.

[26] Wang J. (2024). The Influence of Inclusive Leadership on Employees’ Innovation Performance with the Mediating Effect of Organizational Learning Capacity and Knowledge Sharing in Manufacturing Industry of Guangzhou City, China. *Uniglobal Journal of Social Sciences and Humanities*.

[27] Nishii L. H., Leroy H. (2022). *A Multi-Level Framework of Inclusive Leadership in Organizations*.

[28] Lei H., Saeheng P., Le P. B. (2024). Stimulating Knowledge Sharing Behaviors for Frugal Innovation: The Roles of Inclusive Leadership and Competitive Intensity. *Journal of Knowledge Management*.

[29] Janssens H., Braeckman L., Vlerick P., Van de Ven B., De Clercq B., Clays E. (2018). The Relation between Social Capital and Burnout: A Longitudinal Study. *International Archives of Occupational and Environmental Health*.

[30] Murayama H., Nonaka K., Hasebe M., Fujiwara Y. (2020). Workplace and Community Social Capital and Burnout Among Professionals of Health and Welfare Services for the Seniors: A Multilevel Analysis in Japan. *Journal of Occupational Health*.

[31] El İ. (2019). *The Effects of Psychological Capital and Social Capital on Nurses’ Work Engagement and Burnout*.

[32] Tomlinson M., Jackson D. (2021). Professional Identity Formation in Contemporary Higher Education Students. *Studies in Higher Education*.

[33] Kramer R. M. (2006). Social Identity and Social Capital: The Collective Self at Work. *International Public Management Journal*.

[34] Xu J.-M., Cao M.-G., Gao Q.-C., Lu Y.-X., Stark A. T. (2024). Nurses’ Workplace Social Capital and Sustainable Development: An Integrative Review of Empirical Studies. *Journal of Nursing Management*.

[35] Philippa R., Ann H., Jacqueline M., Nicola A. (2021). Professional Identity in Nursing: A Mixed Method Research Study. *Nurse Education in Practice*.

[36] Veldhuis G. A., Sluijs T., van Zwieten M. H., Bouwman J., Wiezer N. M., Wortelboer H. M. (2020). A Proof-of-Concept System Dynamics Simulation Model of the Development of Burnout and Recovery Using Retrospective Case Data. *International Journal of Environmental Research and Public Health*.

[37] Laschinger H. K. S., Fida R. (2014). A Time-Lagged Analysis of the Effect of Authentic Leadership on Workplace Bullying, Burnout, and Occupational Turnover Intentions. *European Journal of Work & Organizational Psychology*.

[38] Peng W., Li H., Jin D. (2016). A Study on Localization of Inclusive Leadership Structural Dimensions Based on Rootedness Theory. *Human Resources Development of China*.

[39] Tyler D., McCallum R. S. (1998). Assessing the Relationship between Competence and Job Role and Identity Among Direct Service Counseling Psychologists. *Journal of Psychoeducational Assessment*.

[40] Zhang N., Zhang L., Liang Z. (2014). Revision and Evaluation of the Workplace Social Capital Scale. *Chinese Journal of Health Statistics*.

[41] Li C., Shi K., Luo Z. (2001). An Investigation on Job Burnout of Doctor and Nurse. *Chinese Journal of Clinical Psychology*.

[42] Stevens J. (2002). *Applied Multivariate Statistics for the Social Sciences*.

[43] Qin X., Huang Y., Xie Y., Ding F., Ding Q., Fang X. (2024). Effect of Inclusive Leadership of Nursing Managers on Voice Behavior and Innovative Behavior of Department Nurses. *Nursing Practice and Research*.

[44] Liu J., Yuan Y., Ye L., Wang W., Yan F. (2022). Burnout Prevalence and Influencing Factors Among Nurses in a Tertiary General Hospital in Shanghai. *Shanghai Journal of Preventive Medicine*.

[45] Leclerc L., Strenge-McNabb K. K., Thibodeaux T., Campis S., Kennedy K. (2022). Relational Leadership: A Contemporary and Evidence-Based Approach to Improve Nursing Work Environments. *Nursing Management*.

[46] Ahmed F., Xiong Z., Faraz N. A., Arslan A. (2023). The Interplay between Servant Leadership, Psychological Safety, Trust in a Leader and Burnout: Assessing Causal Relationships through a Three-Wave Longitudinal Study. *International Journal of Occupational Safety and Ergonomics*.

[47] Jiang J., Ding W., Wang R., Li S. (2022). Inclusive Leadership and Employees’ Voice Behavior: A Moderated Mediation Model. *Current Psychology*.

[48] Xiaotao Z., Yang X., Diaz I., Yu M. (2018). Is Too Much Inclusive Leadership a Good Thing? An Examination of Curvilinear Relationship Between Inclusive Leadership and Employees’ Task Performance. *International Journal of Manpower*.

[49] Hincapie M. X., Costa P. (2024). Fostering Hybrid Team Performance Through Inclusive Leadership Strategies. *Organizational Dynamics*.

[50] Ke J., Zhang J., Zheng L. (2022). Inclusive Leadership, Workplace Spirituality, and Job Performance in the Public Sector: A Multi-Level Double-Moderated Mediation Model of Leader-Member Exchange and Perceived Dissimilarity. *Public Performance and Management Review*.

[51] Zhang Z., Li Y. (2023). Influencing Factors of Professional Identity, Health Behavior and Their Correlation With Job Burnout in Nursing Staffs in Pension Institution. *American Journal of Health Behavior*.

[52] Shabeer S., Nasir N., Rehman S. (2023). Inclusive Leadership and Career Adaptability: The Mediating Role of Organization-Based Self-Esteem and the Moderating Role of Organizational Justice. *International Journal of Leadership in Education*.

[53] Shafaei A., Nejati M. (2024). Creating Meaningful Work for Employees: The Role of Inclusive Leadership. *Human Resource Development Quarterly*.

[54] Sun B., Fu L., Yan C., Wang Y., Fan L. (2022). Quality of Work Life and Work Engagement Among Nurses With Standardised Training: The Mediating Role of Burnout and Career Identity. *Nurse Education in Practice*.

[55] Cheng C., Bartram T., Karimi L., Leggat S. (2016). Transformational Leadership and Social Identity as Predictors of Team Climate, Perceived Quality of Care, Burnout and Turnover Intention Among Nurses. *Personnel Review*.

[56] Liao R. W., Yeh M. L., Lin K. C., Wang K. Y. (2020). A Hierarchical Model of Occupational Burnout in Nurses Associated with Job-Induced Stress, Self-Concept, and Work Environment. *Journal of Nursing Research*.

[57] Aryankhesal A., Mohammadibakhsh R., Hamidi Y. (2019). Interventions on Reducing Burnout in Physicians and Nurses: A Systematic Review. *Medical Journal of the Islamic Republic of Iran*.

[58] Sartirana M., Currie G., Noordegraaf M. (2019). Interactive Identity Work of Professionals in Management: A Hospital Case Study. *Public Management Review*.

[59] Shakiba Soureh M., Hassani M. (2021). Investigating the Impact of Individual Characteristics on Job Burnout by Examining Mediation Role of Job Stress and Social Capital Among Nurses of Imam Reza Hospital of Urmia. *Quarterly Journal of Nursing Management*.

[60] Zafar S., Raziq M. M., Igoe J., Moazzam M., Ozturk I. (2024). Inclusive Leadership and Innovative Work Behavior: Roles of Autonomous Motivation and Horizontal and Vertical Trust. *Current Psychology*.

[61] Mathuki E., Zhang J. (2024). Cognitive Diversity, Creativity and Team Effectiveness: The Mediations of Inclusion and Knowledge Sharing. *VINE Journal of Information and Knowledge Management Systems*.

[62] Tang Y., Wang Y., Zhou H., Wang J., Zhang R., Lu Q. (2023). The Relationship between Psychiatric Nurses’ Perceived Organizational Support and Job Burnout: Mediating Role of Psychological Capital. *Frontiers in Psychology*.

[63] Liu Y., Aungsuroch Y., Gunawan J., Zeng D. (2021). Job Stress, Psychological Capital, Perceived Social Support, and Occupational Burnout Among Hospital Nurses. *Journal of Nursing Scholarship*.

[64] Adriansyah M. A., Prastika N. D., Muhliansyah (2023). We Are Team: Effectiveness of Team Building Training to Improve Cohesiveness. *International Journal of Professional Business Review*.

[65] Koch T., Denner N. (2022). Informal Communication in Organizations: Work Time Wasted at the Water-Cooler or Crucial Exchange Among Co-workers?. *Corporate Communications: An International Journal*.

[66] Reddy S. M. W., Torphy K., Liu Y. (2021). How Different Forms of Social Capital Created Through Project Team Assignments Influence Employee Adoption of Sustainability Practices. *Organization & Environment*.

[67] McLaney E., Morassaei S., Hughes L., Davies R., Campbell M., Di Prospero L. (2022). A Framework for Interprofessional Team Collaboration in a Hospital Setting: Advancing Team Competencies and Behaviours. *Healthcare Management Forum*.

[68] Hamouche S., Marchand A. (2020). Linking Work, Occupational Identity and Burnout: The Case of Managers. *International Journal of Workplace Health Management*.

[69] Bochatay N. (2018). Individual and Collective Strategies in Nurses’ Struggle for Professional Identity. *Health Sociology Review*.

[70] Xu J., Stark A. T. (2021). A Conceptual Model of Nurses’ Workplace Social Capital: A Theory Synthesis. *BMC Nursing*.

[71] Dall’Ora C., Ball J., Reinius M., Griffiths P. (2020). Burnout in Nursing: A Theoretical Review. *Human Resources for Health*.

